# Assessing the Impact of Nano-Graphene Oxide Addition on Surface Microhardness and Roughness of Glass Ionomer Cements: A Laboratory Study

**DOI:** 10.1155/2024/5597367

**Published:** 2024-06-26

**Authors:** Farahnaz Sharafeddin, Mohammad Mahdi Shirani, Zahra Jowkar

**Affiliations:** ^1^ Department of Operative Dentistry and Biomaterials Research Center School of Dentistry Shiraz University of Medical Sciences, Shiraz, Iran; ^2^ School of Dentistry Shiraz University of Medical Sciences, Shiraz, Iran; ^3^ Department of Operative Dentistry School of Dentistry Shiraz University of Medical Sciences, Shiraz, Iran

## Abstract

**Background:**

Nanomaterials, including nano-graphene oxide (nGO), have emerged as promising modifiers for dental materials. Therefore, this study investigated the effect of incorporating nGO into conventional glass ionomer cement (CGIC) and resin-modified glass ionomer cement (RMGIC) on surface roughness and hardness.

**Methods:**

Sixty disk-shaped specimens (2 × 6 mm) were divided into six groups: CGIC, RMGIC, CGIC with 1 wt.% nGO, CGIC with 2 wt.% nGO, RMGIC with 1 wt.% nGO, and RMGIC with 2 wt.% nGO. Surface roughness (Ra) and Vickers microhardness (VHN) were measured using a surface profilometer and Vickers microhardness tester, respectively. Statistical analysis employed the Kruskal–Wallis and Mann–Whitney tests (*p* <0.05).

**Results:**

The microhardness of RMGICs significantly increased with 1% and 2% nGO (*p*=0.017, *P*=0.001, respectively), while CGICs showed a significant decrease in VHN with nGO incorporation (*p*=0.001). VHN values of all CGIC groups were significantly higher than those of all RMGIC groups (*p*=0.001). Mean surface roughness values for all CGICs were significantly higher than those of RMGIC groups (*p*=0.001). Within the RMGIC groups, mean Ra values of RMGIC + 1 wt.% nGO and RMGIC + 2 wt.% nGO groups decreased significantly compared to the RMGIC control group (*p*=0.001, *p*=0.001, respectively). Among CGIC groups, mean Ra values of 1 wt.% and 2 wt.% nGO/CGIC groups were significantly higher than the CGIC control group (*p*=0.016, *p*=0.001).

**Conclusion:**

Incorporating nGO into RMGICs increased surface microhardness while reducing surface roughness, offering potential advantages for clinical applications. Conversely, adding nGO to CGICs increased surface roughness and decreased surface hardness. These findings emphasize the potential benefits of utilizing nGO in RMGICs and their implications in clinical practice.

## 1. Introduction

Glass poly alkenoate cements, commonly known as glass ionomer cements (GICs), are composed of glass powder and a soluble polymer, which are manually mixed as a powder-liquid system. When water is present, the two components undergo an acid–base reaction to set [1]. These cements are primarily used for restorative purposes such as sealants, luting agents, and cavity bases [2]. GICs are particularly favored for caries prevention in high-risk patients due to their fluoride release [2, 3]. They offer several advantages, such as chemical bonding to the tooth structure, long-term fluoride release against caries, thermal compatibility with teeth, caries prevention, mild pulp response, and tooth-colored appearance [4, 5, 6]. Despite these benefits, GICs have limitations, including sensitivity to moisture during setting, poor aesthetics due to limited translucency, and inferior mechanical properties, which restrict their use in low-stress areas [5, 7]. GICs also exhibit limitations in terms of their resistance to abrasion and wear [8]. In a recent study, the volumetric abrasive wear of two modern GICs, namely a high-viscosity glass ionomer cement (hvGIC; Equia Fil; GC, Tokyo, Japan) and a glass hybrid restorative system (ghRS); Equia Forte; GC, Tokyo, Japan), was examined as potential alternatives to amalgam restorations [8]. The research revealed that both hvGIC and ghRS materials exhibited higher levels of abrasive wear compared to a hybrid composite resin (CR) material [8]. The study also explored the effects of applying a resinous coating to these modern GICs, aiming to enhance their wear resistance. However, the findings suggested that the application of resinous coating did not provide effective protection against abrasive wear in the short or medium term [8]. It was demonstrated that both hvGIC and ghRS were susceptible to abrasive wear, with similar wear rates observed regardless of the presence or absence of a self-adhesive resin containing nanofillers [8]. The available resinous coatings designed to protect the GIC surface were found to have limited durability [8]. Furthermore, all hvGIC and ghRS restorations exhibited significantly higher abrasive wear than the CR material, while the conventional GIC showed inferior wear resistance compared to all other materials tested [8]. Hence, ongoing efforts are being made to improve the surface characteristics of GICs in order to address these limitations.

Various materials have been incorporated into GICs to enhance their mechanical properties [9, 10].

In order to enhance conventional GICs (CGICs), resin-modified GICs (RMGICs) were developed to improve their physical and mechanical characteristics [11]. Including resin materials in RMGICs results in higher compressive and flexural strength than CGICs. Additionally, RMGICs offer the advantage of being partially polymerizable by light in their resin component, allowing clinicians to have some control over the setting reaction [12].

Graphene, a two-dimensional nanosheet with a honeycomb lattice structure, has gained significant attention in various fields. Extensive research on graphene has demonstrated its outstanding physicochemical, optical, and mechanical properties, including biocompatibility, large surface area, high conductivity, suitable electronic transport, excellent mechanical strength, and the potential for integration with adhesive materials and composites to enhance the bond between dentin and resin [13, 14].

Recently, there have been endeavors to enhance the properties of GICs by incorporating graphene-based nanomaterials. The combination of graphene with poly acrylic acid-based glass ionomer cement has shown significant improvements in the mechanical properties of the cement [15]. Among various graphene derivatives, GO is a crucial one, obtained through the oxidative treatment of graphite using Hammer's method [16]. GO contains diverse functional groups, such as ketone, carboxylic, epoxy, and hydroxyl, enabling interactions with organic polymers via van der Waals forces [13]. This property facilitates bonding with different materials, including polymers, biomolecules, DNA, and proteins [16]. Consequently, the compatibility of organic polymers to bond with GO functional groups is enhanced [17]. Studies investigating GO nanofiller's impact on dental restoration's mechanical properties have yielded positive outcomes, demonstrating the beneficial effects of nano-graphene oxide (nGO) nanofillers [14, 18, 19]. A study has reported that GO helps reduce the risk of secondary caries and enhances bond strength [20]. In a prior study, the impact of nGO on the flexural strength of GICs was examined. The findings revealed that incorporating 2% by weight of GO into RMGIC led to an increase in its flexural strength. This finding holds significant clinical significance as it offers a means to enhance the mechanical properties of RMGIC, potentially improving its performance as a dental material [21]. Furthermore, the incorporation of 1% nGO into CGIC and RMGIC resulted in a notable decrease in gingival microleakage for CGIC and both occlusal and gingival microleakage for RMGIC. However, incorporating 2 wt.% nGO did significantly reduce microleakage [22]. Incorporating 1 wt.% and 2 wt.% nGO led to a significant increase in the shear bond strength of CGIC and RMGIC to dentin [10].

Surface finish and quality maintenance are critical characteristics of dental materials, as they minimize undesirable biological interactions and bacterial plaque adhesion [23]. Biofilm, a major contributor to caries and periodontal diseases, can be reduced by minimizing surface roughness. A smooth surface contributes to mechanical durability, aesthetic appearance, optimal optical compatibility with enamel, and glossiness, preventing discoloration and stains [24].

Microhardness, defined as the resistance of materials to plastic deformation, is an important mechanical property that significantly influences the clinical success of dental restorations [3]. There is a correlation between microhardness and resistance to mechanical wear, and it is evident through empirical observation that a greater surface hardness leads to reduced wear volume [8]. Different methods have been suggested to improve surface characteristics such as wear resistance and microhardness such as application of a protective surface coating on GICs [25]. The application of a resin coating considerably enhanced the surface hardness of the recently introduced glass ionomer designed for load-bearing regions (Equia Forte; GC, Tokyo, Japan) in a previous study [25]. Nevertheless, based on a previous study, the application of a resinous coating on GICs does not seem to provide long-lasting and effective protection against severe abrasive wear [8]. Alternative approaches to improve the surface microhardness of GICs include modifying their formulations or integrating fillers into the GIC matrix [9, 25].

The effects of adding nGO to CGIC and RMGIC on their physical and mechanical properties are still uncertain. Previous studies have not specifically examined how including nGO affects the surface microhardness and surface roughness of CGIC and RMGIC. Therefore, considering the potential GO to alter the physical and mechanical properties of GICs, this study aimed to investigate and compare the microhardness and surface roughness of CGIC and RMGIC with and without the incorporation of nGO. The null hypothesis posited that there would be no significant difference in microhardness and surface roughness between CGIC and RMGIC samples with and without the addition of nGO.

## 2. Material and Methods

The study protocol, as outlined in “DOI: dx.doi.org/10.17504/protocols.io.rm7vzxpkrgx1/v1” from protocols.io, received approval from the Research and Ethics Committee of Shiraz University of Medical Sciences (Protocol #IR.SUMS.DENTAL.REC.1402.021).

### 2.1. Sample Preparation

For this experimental study, six groups of disk-shaped specimens were prepared, each consisting of 10 specimens measuring 2 mm in height and 6 mm in diameter.

Before commencing the experiment, the sample size was determined using G Power software (*G* ^*∗*^Power 3.1 software; Heinrich Hein University, Dusseldorf, Germany). The sample size calculation was based on a previous study [26]. In that study, considering a power level of 80% and a significance level of 0.05, the effect size (f) was estimated to be 1.30, with a type I error (*α*) of 0.05 and a type II error (*β*) of 0.2. The results indicated that a minimum of nine specimens per subgroup would be required for the present study. Hence, a sample size of 10 was selected for each study group.

**Figure 1:**
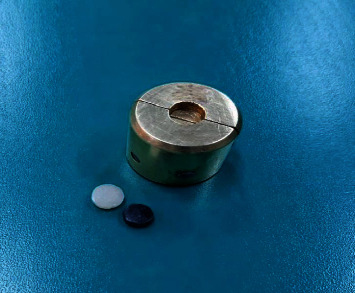
Mold used in the study for specimen preparation.

A cylindrical brass mold with a central gap (Figure 1) was used for specimen preparation. The molds were filled using a vibrator machine (Denstar-500; Denstar, Daegu, South Korea) for 15 s per sample to prevent the formation of air bubbles. The materials utilized in this study are presented in Table 1.

A total of 60 samples were prepared and divided into the following groups (*n* = 10) for CGIC and RMGIC:

1. Group 1: CGIC samples (control group).
2. Group 2: CGIC samples with 1% nGO.
3. Group 3: CGIC samples with 2% nGO.
4. Group 4: RMGIC samples (control group).
5. Group 5: RMGIC samples with 1% nGO.
6. Group 6: RMGIC samples with 2% nGO.

Group 1 (CGIC control group): In this group (*n* = 10), one scoop of powder and one drop of liquid from a control CGIC (GC Gold label 2; GC, Tokyo, Japan) were mixed on a cold glass slab using a plastic spatula for 25 s, following the manufacturer's instructions. The cylindrical brass molds were then overfilled with the mixture, and a transparent mylar matrix strip (Fintrec Transparent Matrix; Pulpdent, Watertown, MA, USA) was placed on the top surface of the mold until the CGIC was completely set (5.5 min from the start of mixing).

Group 2 (CGIC + 1% nGO): In group 2 (*n* = 10), the specimens consisted of 99 wt.% CGIC and 1 wt.% of nGO (Graphene; Knvindia, Maharashtra, India). The CGIC (99 wt.%) and nGO (1 wt.%) were accurately weighed using a weighing machine (A and D, GR + 360, Tokyo, Japan) with a resolution of 0.0001 g. Subsequently, the nGO powder was added to the CGIC powder and mixed in an amalgamator (FD-5000-A; Faghihi, Tehran, Iran) using clean amalgam capsules for 20s to obtain a uniform mixture. The specimens were then prepared following the same procedure described for Group 1.

Group 3 (CGIC + 2 wt.% nGO): In this group (*n* = 10), the specimens contained 98 wt.% CGIC and 2 wt.% of nGO. The specimens were mixed and prepared following the same procedure as described for Groups 1 and 2.

Group 4 (RMGIC control group): In Group 4 (*n* = 10), the disks were prepared using one scoop of a control RMGIC powder (Fuji II LC Gold A2; GC, Tokyo, Japan), which was mixed with two drops of liquid for 25 s according to the manufacturer's instructions, with a powder-to-liquid ratio of 3.2 : 1 by weight. The molds were overfilled, as mentioned before, and a mylar matrix strip was placed on the upper surface of the mold. The upper surface of each sample was then light-cured for 40 s using an LED light-curing unit (Blue Lex LD-105; Monitex, Taipei, Taiwan) with a light intensity of 1,200 mW/cm^2^, following the manufacturer's directions. The curing tip was positioned 1 mm away from the upper surface of the sample. After the initial setting of the specimens, they were removed from the mold.

Group 5 (RMGIC + 1 wt.% nGO): Group 5 (*n* = 10) comprised specimens consisting of 99 wt.% RMGIC and 1 wt.% of nGO. The samples were prepared and evaluated following the same procedure described for Group 4.

Group 6 (RMGIC + 2 wt.% nGO): Group 6 (*n* = 10) consisted of specimens containing 98 wt.% RMGIC and 2 wt.% of nGO. The samples were prepared and evaluated following the same procedure described for Groups 4 and 5.

**Figure 2:**
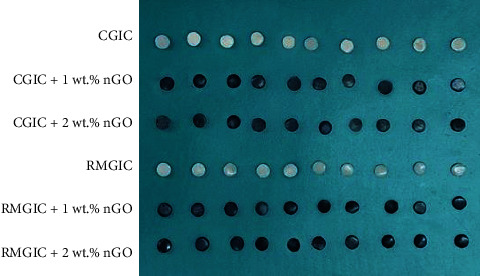
Specimens prepared for the study in different groups.

After the specimens were completely set, they were removed from the mold and coated with a thin layer of copal varnish (Kimia Varnish; Kimia, Tehran, Iran) to protect against moisture. Subsequently, all samples were stored in an incubator at 100% humidity and 37°C for 24 hr. The samples were then polished using a low-speed handpiece (Ti-Max X25L; NSK, Tochigi, Japan) and polishing discs (Polishing discs, TOR VM, Moscow, Russia) with different grits. The polishing process was performed for 60 s on the upper surface of each disk. The specimens were washed with distilled water using an ultrasonic bath (Easyclean; Renfert, Hilzingen, Germany) for 1 min to eliminate surface debris. Following that, the specimens underwent microhardness and surface roughness tests. The prepared specimens can be observed in Figure 2.

Surface roughness (Ra, *μ*m) measurements were taken at three distinct points on the upper surface of each specimen. A profilometer (Rugosurf 20; TESA Technology, Renens, Switzerland), calibrated before the evaluation, was used to measure Ra. The profilometer was in contact with the specimen's surface, and the stylus was moved across the designated points to capture the surface profile information. The profilometer provided precise measurements of the surface irregularities, allowing the determination of Ra values. Three replicate readings were taken at each selected point on the specimen's surface to ensure the accuracy of the measurements. Subsequently, the arithmetic mean of these three measurements was calculated to obtain the representative Ra value for each specimen.

**Figure 3:**
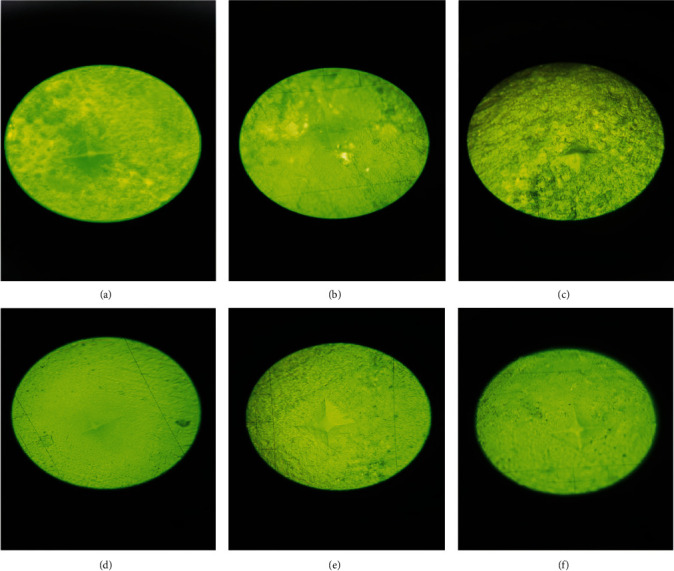
Indentation surface microhardness in all study groups. Panels (a–f) represent the following groups: (a) conventional glass ionomer cement (CGIC), (b) CGIC + 1 wt.% nano-graphene oxide (nGO), (c) CGIC + 2 wt.% nGO, (d) RMGIC, (e) RMGIC + 1 wt.% nGO, and (f) RMGIC + 2 wt.% nGO.

A digital Vickers microhardness testing device (HXD-1000TMC; Taiming Optical Instrument, Shanghai, China) with a force of 300 g/15 s was employed to conduct the hardness test on the upper surfaces of the specimens at three points. The mean values were recorded as the VHN level of each sample. The distance between each indentation point was at least 1 mm. The indentation surface can be seen in Figure 3.

The data obtained from the experiments were analyzed using the Statistical Package for the Social Sciences (SPSS) software for Windows version 20.0 (IBM SPSS software; SPSS, Chicago, IL, USA). The Shapiro–Wilk test was conducted to evaluate the normality of the data. Afterward, the Kruskal–Wallis and Mann–Whitney tests were utilized to evaluate the impact of glass ionomer type and nGO percentage on the surface roughness and hardness of the samples (*P*  < 0.05).

## 3. Results

In this experimental investigation, the Kruskal–Wallis test and Mann–Whitney test revealed a notable reduction in the mean Vickers microhardness values (VHN) in the 1% and 2% nGO/CGIC groups compared to the control group (71.19 ± 2.99) (*p*=0.001). The mean VHN values in the 1% and 2% nGO/CGIC groups were 59.33 ± 4.18 and 57.53 ± 2.77, respectively. However, no significant distinction was observed between the 1% nGO/CGIC and 2% nGO/CGIC groups (*p*=0.199). The mean VHN value in the 1% nGO/RMGIC group was 44.41 ± 4.88, higher than the control group (38.59 ± 4.83) (*p*=0.017). Similarly, the mean VHN value in the 2% nGO/RMGIC group was 48.01 ± 2.09, also higher than the control group (*p*=0.001). Furthermore, the VHN value of the 2% nGO/RMGIC group exhibited a higher value than the 1% nGO/RMGIC group (*p*=0.016). Additionally, the VHN values of all CGIC groups were significantly greater than the VHN values of all RMGIC groups (*p*=0.001).

Based on the results obtained from the Kruskal–Wallis test and Mann–Whitney test, it was determined that the mean surface roughness values (Ra *µ*m) of all CGICs were significantly higher than those of the RMGIC groups (*p*=0.001).

Among the CGIC groups, the mean Ra values of the 1% and 2% GO/CGIC groups were significantly higher than the CGIC control group (0.947 ± 0.12) (*p*=0.016, *p*=0.001, respectively). The mean surface roughness values in the 1% and 2% nGO/CGIC groups were 2.216 ± 0.3 and 2.443 ± 0.24, respectively. However, no significant difference was observed between the CGIC + 1% nGO and CGIC + 2% nGO groups (*p*=0.151).

Within the RMGIC groups, a significant decrease in the mean Ra values was observed in the RMGIC + 1% nGO (0.606 ± 0.16) and RMGIC + 2% nGO (0.568 ± 0.12) groups compared to the RMGIC control group (0.781 ± 0.11) (*p*=0.001, *p*=0.001, respectively). Nevertheless, no significant difference was found between the RMGIC + 1% nGO and RMGIC + 2% nGO groups (*p*=0.326).

## 4. Discussion

Based on the findings of the present study, the null hypothesis was rejected because significant differences in microhardness and surface roughness between CGIC and RMGIC samples with and without the addition of nGO were observed in the present study.

Surface hardness is a critical characteristic of dental materials and indicates the clinical performance of restorations. It refers to the material's ability to resist permanent indentation or penetration [27]. Reduced microhardness can lead to degradation of the restorative material, resulting in increased surface roughness, plaque accumulation, color changes, and loss of anatomical form in clinical settings [28]. Therefore, even modern GIC materials such as glass hybrid restorative systems and high-viscosity GICs do not comply with the current clinical requirements [8]. Therefore, efforts are being made to improve the surface characteristics of GICs.

In an experimental study by Sari et al. [29]the authors investigated the impact of incorporating glass fibers and GO into an RMGIC. They prepared experimental RMGICs by adding varying weight percentages of glass fibers (5%, 10%, and 20%) and GO (1%, 3%, and 5%) to the RMGIC powder. The study examined surface roughness, flexural strength, Vickers microhardness, water sorption, and solubility of the modified RMGICs. The results indicated that adding GO to RMGIC increased the Vickers microhardness. Furthermore, incorporating both fibers and GO into RMGIC resulted in higher surface roughness and flexural strength [29]. In another study by Sharafeddin et al. [21] it was concluded that adding 2 wt.% nGO to RMGIC enhanced the flexural strength of RMGICs [21]. These findings suggest that reinforcing RMGICs with nGO could improve their mechanical properties. Consistent with previous studies, the present study observed a significant increase in microhardness when 1 wt.% and 2 wt.% nGO were added to the RMGIC control group.

GO, a graphene derivative, is a biocompatible material that contains oxygen-containing groups and can be utilized to enhance the mechanical properties of scaffolds or nanocomposites. Unlike graphene, GO tends to be hydrophilic due to the presence of functional groups such as carboxyl, hydroxyl, and epoxy groups. Similar to the carboxylic acid found in the GIC liquid, these functional groups contribute to GO's hydrophilicity. The presence of functional groups in nGO also reinforces RMGIC and its compatibility with polar solvents allows for effective dispersion. Adding nGO to RMGIC improves the dispersion at the molecular level and enhances interfacial interaction, resulting in increased microhardness [30]. In the present study, the inclusion of nGO may have facilitated the formation of a polymer matrix between the constituents of RMGIC, leading to an improvement in Vickers microhardness. Additionally, the observed increase in microhardness of RMGIC could be attributed to the inherent high strength of nGO [30].

The combination of poly methyl methacrylate (PMMA) and nGO has been shown to enhance the mechanical properties of PMMA resin [31]. When incorporated into a matrix, nGO plays a crucial role in crack deflection and bridging, thus improving the mechanical properties of PMMA [32]. GO's ability to exhibit crack bridging, pulling out, crack deflection, and crack tip shielding could enhance the mechanical characteristics of RMGICs, including microhardness, which may explain the results obtained in the current study.

One of the primary drawbacks of using graphene as a reinforcement material is its limited ability to disperse uniformly within a matrix, which hinders its effectiveness as a mechanical reinforcement. Graphene's hydrophobic nature and strong van der Waals attraction cause the sheets to aggregate into weakly interacting monolayers, thereby affecting the enhancement of the matrix [33, 34]. In contrast to the RMGIC groups, our results demonstrated a significant decrease in Vickers microhardness when nGO was added to CGICs. Unlike previous studies, we mechanically combined nGO and CGIC without employing an intermediate chemical compound to stabilize their linkage. This approach reduced the likelihood of achieving a homogeneous mixture of CGIC/nGO. We observed agglomeration of nGO particles during the mixing of powdered CGIC/GO with liquid, which potentially compromised the mechanical properties of the CGIC/nGO hybrid. The CGIC samples in our study exhibited a significant decrease in microhardness by adding 1 wt.% and 2 wt.% GO. This hybrid may require more working time to achieve homogeneity and improve microhardness.

Furthermore, the nGO powder incorporated into the GIC powder did not react with the GIC due to its insolubility, contributing to decreased microhardness values. Considering the results of the current study, although the microhardness of CGIC with 2% nGO was slightly lower than that of CGIC with 1% nGO, this difference was not statistically significant. We concluded that as the percentage of nGO increased, a portion of the added graphene bonded with the functional groups of the GIC, preventing further reduction in strength and microhardness.

RMGICs utilize the same glass components as CGICs. The acidic polymer may also be the same, although, in some materials, it is modified with unsaturated vinyl groups at the end of side chains. These vinyl-modified polyalkenoic acids can participate in the addition polymerization reaction, forming covalent cross-links between the polymer chains [1]. RMGICs undergo a setting reaction that involves an acid–base reaction similar to CGICs. Additionally, they undergo a polymerization reaction involving the unsaturated side chains on the modified polyacid. In certain RMGICs, the networks formed by the polyacid and ionically cross-linked polyalkenoate chains provide the structural integrity of the cement [35]. The advantages of RMGICs include shorter setting time, reduced sensitivity to moisture during the early stages, extended working time, and improved strength properties compared to CGICs [36]. By adding 1 wt.% and 2 wt.% nGO to RMGIC, more polymerization reactions may occur between the vinyl-modified polyalkenoic acid of RMGIC and the functional groups of nGO, which are similar to those in CGIC. This reaction increases the working time and enhances the strength of RMGIC/nGO compared to RMGIC. Notably, in our study, the RMGIC/nGO mixture did not exhibit agglomerated nGO particles during mixing with the liquid (attributed to the increased working time), resulting in increased microhardness.

In our study, the microhardness of RMGIC increased significantly when 2 wt.% GO was added compared to 1 wt.% GO. This finding suggests that the involvement of more vinyl groups, facilitated by the increased nGO content, leads to more polymerization reactions, as mentioned earlier, resulting in a significant increase in microhardness.

Consistent with previous studies, the microhardness values of all CGICs in our study were significantly higher than those of RMGICs [27, 37]. This difference in microhardness may be attributed to the resin matrix hydroxyethylmethacrylate (HEMA) in CGIC, which affects GIC microhardness in two ways. First, the cross-linkage formed between carboxylate chains in GIC and HEMA molecules disrupts the formation of the typical cross-linking mechanism through Ca^2+^ ions, leading to the separation of polyacid chains from each other [38]. Second, the water absorbed by the resin matrix (HEMA) may hinder the secondary curing reaction on the cement's surface layer [27, 38].

Surface roughness plays a crucial role in clinical settings, and various methods can be used to evaluate it, including visual evaluation, scanning electron microscopy, and profilometry analysis [39]. Our study employed profilometry analysis to measure Ra values due to its precision, adaptability, and ease of use.

Particle size has been reported to influence surface roughness in GICs, with smaller particle sizes associated with better polishability [39, 40]. CGICs generally have larger particle sizes than RMGICs, and SEM evaluations have shown that CGICs exhibit higher surface voids than RMGICs [40]. In line with previous findings, all CGIC groups in our study, regardless of the percentage of added graphene, had higher Ra values than the RMGIC groups. This result can be attributed to the smaller particle size of RMGICs, resulting in a smoother surface, and the presence of resin, which improves surface structure and polishability, leading to lower Ra values [40]. During the study, the specimens were polished to simulate clinical conditions, and the smoother surface of RMGIC groups with fewer voids contributed to their lower Ra values compared to the CGIC-containing groups.

Furthermore, adding 1 wt.% and 2 wt.% nGO powder to RMGIC groups in our study significantly decreased surface roughness compared to the control group. The hydroxyl groups in nGO can form hydrogen bonds with the hydroxyl groups in HEMA and the carboxylic acid groups in poly acrylic acid. This chemical bonding can enhance the mechanical properties of GICs by reducing the interfacial tension between the GIC components [41]. Additionally, the large specific surface area and rough surface of nGO contribute to improved matrix adhesion and interlocking [30]. The decrease in surface roughness observed by adding 1% and 2% nGO in our study may be attributed to the chemical bonding of nGO with RMGICs, promoting better particle integration and the formation of a homogeneous mixture.

GO is widely recognized for suppressing nanoscale crack propagation in matrices [42]]. The previous study on crack branching mechanisms can also help explain the surface roughness improvement caused by nGO. This mechanism involves four main aspects: crack bridging, pull-out, crack deflection, and crack tip shielding [43]. In crack bridging, nGO acts as a bridge between opposing surfaces, reducing the force of crack propagation. Pull-out occurs when the shear force exceeds the interface strength, causing the graphene and matrix to stick together. The two-dimensional nanosheet structure of graphene leads to tortuous crack propagation paths, dissipating more energy through crack deflection. When cracks propagate to graphene sheets, their propagation becomes confined to the vicinity of the graphene sheet due to insufficient energy [44]. The present study showed that the RMGIC matrix cracks are expected to be arrested and bridged by nGO, contributing to its improvement.

In contrast to the RMGIC groups, adding nGO to CGIC groups significantly increased surface roughness. This finding can be attributed to preventing the integration and entanglement of powder particles and liquid components by graphene, which lacks hydrogen bond formations. The liquid component of GICs also plays a role in surface roughness, and efforts have been made to reduce its viscosity by removing polyacid and adding it to the powder [40]. RMGICs, compared to CGICs, contain methacrylate components common in resin composites and a liquid portion with a water-miscible methacrylate monomer. RMGICs also contain free radical initiators that initiate an additional chain reaction during the curing process, unlike CGICs, which rely solely on the acid–base reaction [45, 46]. Consequently, CGICs have different micromorphology, and adding nGO components to CGICs results in more complex manipulation of powder and liquid, leading to difficulties in mixing them and resulting in more voids and higher surface roughness.

No significant difference in surface roughness was observed between the 1 wt.% nGO/CGIC and 2 wt.% nGO/CGIC groups in both CGICs and RMGICs. It seems that while the addition of 1 wt.% and 2 wt.% nGO by weight increases the surface roughness of CGICs and decreases the surface roughness of RMGICs, further increasing the weight percentage of nGO beyond a specific amount does not have a substantial effect on changing the surface roughness.

Among the limitations of this study, it is essential to note that it was conducted as an in vitro study, lacking the reconstruction of various clinical conditions within the oral environment. Factors such as the impact of food acidity, chewing forces, and brushing forces were not considered. Therefore, the findings of this study may not fully represent the complete effect of nGO incorporation in real-life scenarios.

It is suggested that further studies be conducted to address these limitations and obtain more comprehensive results. These future investigations should focus on exploring different concentrations of GO and evaluating additional mechanical and physical properties of GICs when incorporated with nGO. This approach would contribute to achieving more reliable and comprehensive outcomes, providing a broader understanding of the potential benefits and applications of nGO in GICs.

## 5. Conclusion

In conclusion, the findings of this study contribute to the growing body of knowledge on nGO-reinforced GICs, providing valuable insights for researchers and clinicians in pursuing advanced dental materials with improved mechanical properties and clinical outcomes. Based on the current study's limitations, although adding nGO to RMGIC can be desirable in a clinical setting, as demonstrated in this study, adding nGO to CGIC has resulted in increased surface roughness. However, the surface hardness of CGIC was increased following the incorporation of nGO into CGIC compared to RMGIC, which is clinically desirable.

## Figures and Tables

**Table 1:** Materials used in this study.

Material	Country	Company	Composition	LOT number
Conventional glass ionomer cement (CGIC) (GC Gold label 2)	Japan	GC	Powder: fluoroaluminosilicate glass Liquid: poly acrylic acid, itaconic acid, tartaric acid, maleic acid, water	22211221

Resin-modified glass ionomer cement (RMGIC) (Fuji II LC Gold A2)	Japan	GC	Powder: fluoroaluminosilicate glass Liquid: poly acrylic acid, urethane dimethacrylate, comphorquinone, distilled water, 2-hydroxy ethyl methacrylate	2209031

Nano-graphene oxide (nGO) (graphene)	India	Knvindia	Graphene oxide	—

Varnish	Iran	Kimia	Copal resin, ethanol	44020307

## Data Availability

The data that support the findings of this study are available on request from the corresponding author.
